# 2-[1-(4-Chloro­benzo­yl)pyrrolidin-2-yl]-4,4,5,5-tetra­methyl-4,5-dihydro­imidazole-1-oxyl-3-oxide

**DOI:** 10.1107/S1600536811001462

**Published:** 2011-01-15

**Authors:** Min Tian, Zhuo Xiang, Si-Yuan Zhou, Lin-Lin Jing, Hai-Bo Wang, Xiao-Li Sun

**Affiliations:** a Department of Chemistry, School of Pharmacy, Fourth Military Medical University, Changle West Road 17, 710032 Xi-An, People’s Republic of China; b Department of Pharmaceutics, School of Pharmacy, Fourth Military Medical University, Changle West Road 17, 710032 Xi-An, People’s Republic of China; c Department of Pharmacy, Lanzhou General Hospital, Lanzhou Command, Lanzhou 730050, People’s Republic of China

## Abstract

In the title compound, C_18_H_23_ClN_3_O_3_, the imidazole ring system has an envelope conformation, whereas the nitronyl nitroxide unit displays a half-chair or twisted conformation. In the crystal, C—H⋯O hydrogen bonds build up a three-dimensional network.

## Related literature

For the biological activity of nitronyl nitroxides, see: Soule *et al.* (2007 ▶ and for their coordination properties, see: Masuda *et al.* (2009 ▶). For related structures, see: Iqbal *et al.* (2009 ▶); Wang *et al.* (2009 ▶). For puckering parameters, see: Cremer & Pople (1975 ▶).
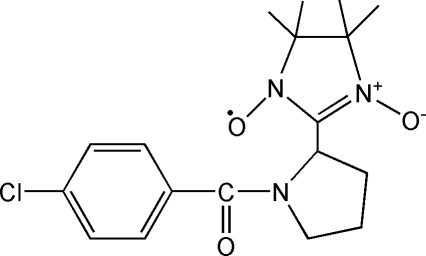


## Experimental

### 

#### Crystal data

C_18_H_23_ClN_3_O_3_
*M* *_r_* = 364.84
Monoclinic, 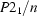

*a* = 11.6202 (19) Å
*b* = 8.2694 (13) Å
*c* = 20.315 (3) Å
β = 105.636 (2)°
*V* = 1879.8 (5) Å^3^
*Z* = 4
Mo *K*α radiation
μ = 0.23 mm^−1^
*T* = 296 K
0.34 × 0.29 × 0.18 mm

#### Data collection

Bruker APEXII CCD diffractometer
Absorption correction: multi-scan (*SADABS*; Sheldrick, 2008*a* ▶) *T*_min_ = 0.928, *T*_max_ = 0.960
9089 measured reflections
3342 independent reflections
1791 reflections with *I* > 2σ(*I*)
*R*_int_ = 0.051

#### Refinement

*R*[*F* ^2^ > 2σ(*F* ^2^)] = 0.051
*wR*(*F* ^2^) = 0.152
*S* = 1.03
3342 reflections
230 parameters
H-atom parameters constrained
Δρ_max_ = 0.19 e Å^−3^
Δρ_min_ = −0.18 e Å^−3^

### 

Data collection: *APEX2* (Bruker, 2007 ▶); cell refinement: *SAINT* (Bruker, 2007 ▶); data reduction: *SAINT*; program(s) used to solve structure: *SHELXS97* (Sheldrick, 2008*b* ▶); program(s) used to refine structure: *SHELXL97* (Sheldrick, 2008*b* ▶); molecular graphics: *ORTEPIII* (Burnett & Johnson, 1996 ▶) and *ORTEP-3 for Windows* (Farrugia, 1997 ▶); software used to prepare material for publication: *SHELXTL* (Sheldrick, 2008*b* ▶) and *PLATON* (Spek, 2009 ▶).

## Supplementary Material

Crystal structure: contains datablocks I, global. DOI: 10.1107/S1600536811001462/dn2639sup1.cif

Structure factors: contains datablocks I. DOI: 10.1107/S1600536811001462/dn2639Isup2.hkl

Additional supplementary materials:  crystallographic information; 3D view; checkCIF report

## Figures and Tables

**Table 1:** Hydrogen-bond geometry (Å, °)

*D*—H⋯*A*	*D*—H	H⋯*A*	*D*⋯*A*	*D*—H⋯*A*
C2—H2*A*⋯O2^i^	0.93	2.54	3.458 (4)	170
C3—H3*A*⋯O1^i^	0.93	2.45	3.314 (4)	154
C8—H8*A*⋯O3^ii^	0.97	2.44	3.101 (4)	125

Symmetry codes: (i) 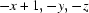; (ii) 
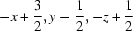
.

**Fig. 1.**
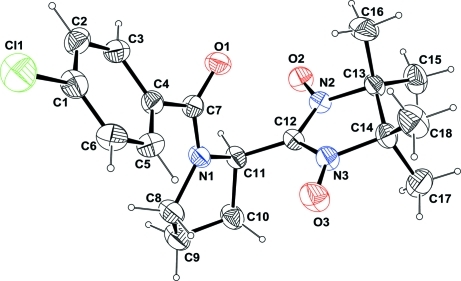
Molecular structure of the title compound (I), showing the atom labeling scheme. Displacement ellipsoids are drawn at the 30% probability level. H atoms are represented as small spheres of arbitrary radii.

## supplementary crystallographic information

### Comment

Nitronyl nitroxides, stable organic radicals, display interesting properties in many fields as magnetism, anticancer, antiradiation and antioxidation *etc* (Soule *et al.*, 2007). Nitronyl nitroxides have received considerable attention recently (Iqbal *et al.*, 2009; Wang *et al.*, 2009). The title compound can also be used for coordination with many metal cations, such as Mn^2+^, Cu^2+^ and Ni^2+^ leading to some molecule based magentic materials (Masuda, *et al.*, 2009).

In the title compound, the indole ring system has an envelope conformation with puckering parameters Q(2)= 0.370 (4)Å and φ= 78.0 (5)° (Cremer & Pople, 1975) whereas the nitronyl nitroxide moiety displays a half-chair or twisted conformation with Q(2)= 0.228 (3)Å and φ= 308.6 (7)°. Occurence of C-H···O hydrogen bonds build up a three dimensional network (Table 1).

### Experimental

2,3-Dimethyl-2,3-bis(hydroxylamino) butane (1.48 g, 10.0 mmol) and 1-(4-chlorobenzoyl)pyrrolidine-2-carbaldehyde (2.38 g, 10.0 mmol) were dissolved in methanol. The reaction was stirred for 10 h at reflux temperature, then cooled to room temperature and filtered. The cake was suspended in dichloromethane (150.0 ml) and cooled at ice bath for 10 min,Then the reaction mixture was added to an aqueous solution of NaIO_4_ and stirred for 15 min to give a amaranthine solution. The aqueous phase was extracted with CH_2_Cl_2_ and the organic layer was combined and dried over MgSO_4_. Then the solvent was removed to give a amaranthine residue which was purified by a flash column chromatography with the elution of *n*-hexane/ethyl acetate (1:1) to yield the title compound (I) as a dark amaranthine powder. Single crystals of compound (I) were obtained from the 1/1 mixed solution of *n*-heptane and dichloromethane.

### Refinement

All H atoms attached to C atoms were fixed geometrically and treated as riding with C—H = 0.96 Å (methyl), 0.97 Å (methylene) and 0.93 Å (aromatic) with U_iso_(H) = 1.2U_eq_(C) or U_iso_(H) = 1.5U_eq_(Cmethyl).

### Crystal data

**Table:**

C_18_H_23_ClN_3_O_3_	*F*(000) = 772
*M**_r_* = 364.84	*D*_x_ = 1.289 Mg m^−^^3^
Monoclinic, *P*2_1_/*n*	Mo *K*α radiation, λ = 0.71073 Å
Hall symbol: -P 2yn	Cell parameters from 1006 reflections
*a* = 11.6202 (19) Å	θ = 2.3–19.1°
*b* = 8.2694 (13) Å	µ = 0.23 mm^−^^1^
*c* = 20.315 (3) Å	*T* = 296 K
β = 105.636 (2)°	Block, purple
*V* = 1879.8 (5) Å^3^	0.34 × 0.29 × 0.18 mm
*Z* = 4	

### Data collection

**Table:**

Bruker APEXII CCD diffractometer	3342 independent reflections
Radiation source: fine-focus sealed tube	1791 reflections with *I* > 2σ(*I*)
graphite	*R*_int_ = 0.051
φ and ω scans	θ_max_ = 25.1°, θ_min_ = 1.8°
Absorption correction: multi-scan (*SADABS*; Sheldrick, 2008a)	*h* = −13→13
*T*_min_ = 0.928, *T*_max_ = 0.960	*k* = −9→5
9089 measured reflections	*l* = −24→22

### Refinement

**Table:**

Refinement on *F*^2^	Primary atom site location: structure-invariant direct methods
Least-squares matrix: full	Secondary atom site location: difference Fourier map
*R*[*F*^2^ > 2σ(*F*^2^)] = 0.051	Hydrogen site location: inferred from neighbouring sites
*wR*(*F*^2^) = 0.152	H-atom parameters constrained
*S* = 1.03	*w* = 1/[σ^2^(*F*_o_^2^) + (0.0548*P*)^2^] where *P* = (*F*_o_^2^ + 2*F*_c_^2^)/3
3342 reflections	(Δ/σ)_max_ < 0.001
230 parameters	Δρ_max_ = 0.19 e Å^−^^3^
0 restraints	Δρ_min_ = −0.18 e Å^−^^3^

### Special details

**Table:**

Geometry. All esds (except the esd in the dihedral angle between two l.s. planes) are estimated using the full covariance matrix. The cell esds are taken into account individually in the estimation of esds in distances, angles and torsion angles; correlations between esds in cell parameters are only used when they are defined by crystal symmetry. An approximate (isotropic) treatment of cell esds is used for estimating esds involving l.s. planes.
Refinement. Refinement of *F*^2^ against ALL reflections. The weighted *R*-factor *wR* and goodness of fit *S* are based on *F*^2^, conventional *R*-factors *R* are based on *F*, with *F* set to zero for negative *F*^2^. The threshold expression of *F*^2^ > σ(*F*^2^) is used only for calculating *R*-factors(gt) *etc*. and is not relevant to the choice of reflections for refinement. *R*-factors based on *F*^2^ are statistically about twice as large as those based on *F*, and *R*- factors based on ALL data will be even larger.

### Fractional atomic coordinates and isotropic or equivalent isotropic displacement parameters (Å^2^)

**Table:**

	*x*	*y*	*z*	*U*_iso_*/*U*_eq_	
Cl1	0.24418 (8)	−0.02934 (14)	0.22799 (5)	0.0934 (4)	
O1	0.61663 (17)	0.1756 (3)	0.04529 (10)	0.0602 (6)	
O2	0.84803 (18)	0.2887 (2)	−0.03089 (10)	0.0613 (6)	
O3	0.7771 (2)	0.5304 (3)	0.15800 (11)	0.0793 (8)	
N1	0.7572 (2)	0.1723 (3)	0.14502 (11)	0.0496 (6)	
N2	0.81945 (18)	0.3955 (3)	0.00771 (11)	0.0462 (6)	
N3	0.7868 (2)	0.5102 (3)	0.09729 (12)	0.0522 (7)	
C1	0.3608 (3)	0.0241 (4)	0.19376 (17)	0.0586 (9)	
C2	0.3684 (3)	−0.0441 (4)	0.13384 (17)	0.0601 (9)	
H2A	0.3113	−0.1182	0.1111	0.072*	
C3	0.4613 (3)	−0.0021 (3)	0.10737 (15)	0.0537 (8)	
H3A	0.4661	−0.0471	0.0662	0.064*	
C4	0.5484 (2)	0.1070 (3)	0.14140 (14)	0.0472 (7)	
C5	0.5373 (3)	0.1743 (4)	0.20168 (15)	0.0561 (8)	
H5	0.5942	0.2480	0.2250	0.067*	
C6	0.4434 (3)	0.1341 (4)	0.22772 (16)	0.0609 (9)	
H6	0.4362	0.1812	0.2680	0.073*	
C7	0.6433 (3)	0.1542 (3)	0.10742 (15)	0.0479 (7)	
C8	0.8104 (3)	0.1429 (4)	0.21806 (14)	0.0617 (9)	
H8A	0.7748	0.0491	0.2335	0.074*	
H8B	0.8008	0.2360	0.2451	0.074*	
C9	0.9400 (3)	0.1137 (5)	0.22247 (16)	0.0697 (10)	
H9A	0.9537	0.0020	0.2121	0.084*	
H9B	0.9904	0.1401	0.2676	0.084*	
C10	0.9636 (3)	0.2265 (4)	0.16896 (16)	0.0635 (9)	
H10A	1.0315	0.1900	0.1537	0.076*	
H10B	0.9787	0.3357	0.1866	0.076*	
C11	0.8480 (2)	0.2188 (4)	0.11055 (14)	0.0486 (8)	
H11	0.8553	0.1332	0.0786	0.058*	
C12	0.8187 (2)	0.3736 (4)	0.07277 (13)	0.0438 (7)	
C13	0.7659 (3)	0.5550 (4)	−0.01959 (15)	0.0510 (8)	
C14	0.7768 (3)	0.6477 (4)	0.04813 (15)	0.0531 (8)	
C15	0.8360 (3)	0.6285 (4)	−0.06555 (17)	0.0779 (11)	
H15A	0.9181	0.6412	−0.0402	0.117*	
H15B	0.8029	0.7322	−0.0816	0.117*	
H15C	0.8311	0.5585	−0.1039	0.117*	
C16	0.6380 (3)	0.5167 (4)	−0.05963 (18)	0.0816 (12)	
H16A	0.6388	0.4451	−0.0967	0.122*	
H16B	0.5976	0.6150	−0.0775	0.122*	
H16C	0.5971	0.4657	−0.0300	0.122*	
C17	0.8923 (3)	0.7448 (4)	0.07346 (18)	0.0801 (11)	
H17A	0.8999	0.7809	0.1193	0.120*	
H17B	0.8901	0.8367	0.0443	0.120*	
H17C	0.9593	0.6777	0.0727	0.120*	
C18	0.6703 (3)	0.7508 (5)	0.05121 (19)	0.0856 (12)	
H18A	0.5987	0.6872	0.0375	0.128*	
H18B	0.6643	0.8415	0.0210	0.128*	
H18C	0.6807	0.7887	0.0971	0.128*	

### Atomic displacement parameters (Å^2^)

**Table:**

	*U*^11^	*U*^22^	*U*^33^	*U*^12^	*U*^13^	*U*^23^
Cl1	0.0622 (6)	0.1224 (10)	0.1045 (8)	−0.0041 (5)	0.0375 (6)	0.0165 (6)
O1	0.0588 (13)	0.0744 (16)	0.0446 (13)	−0.0067 (11)	0.0091 (10)	0.0026 (10)
O2	0.0730 (14)	0.0614 (16)	0.0543 (13)	0.0069 (11)	0.0252 (11)	−0.0072 (11)
O3	0.114 (2)	0.0787 (18)	0.0516 (14)	0.0164 (14)	0.0341 (13)	−0.0040 (12)
N1	0.0472 (15)	0.0576 (17)	0.0428 (14)	−0.0007 (12)	0.0099 (12)	0.0103 (11)
N2	0.0456 (13)	0.0504 (16)	0.0447 (15)	0.0033 (12)	0.0155 (11)	−0.0006 (12)
N3	0.0622 (16)	0.0504 (17)	0.0464 (16)	0.0085 (12)	0.0185 (13)	0.0005 (13)
C1	0.0430 (18)	0.068 (2)	0.065 (2)	0.0065 (16)	0.0152 (16)	0.0131 (18)
C2	0.0481 (19)	0.052 (2)	0.075 (2)	−0.0038 (15)	0.0087 (17)	0.0014 (17)
C3	0.0515 (19)	0.053 (2)	0.0521 (19)	0.0012 (16)	0.0069 (16)	−0.0061 (15)
C4	0.0467 (17)	0.0432 (19)	0.0498 (18)	0.0038 (14)	0.0100 (14)	0.0042 (14)
C5	0.0522 (19)	0.059 (2)	0.055 (2)	−0.0026 (15)	0.0107 (16)	−0.0082 (16)
C6	0.055 (2)	0.075 (3)	0.0533 (19)	0.0059 (18)	0.0165 (16)	−0.0048 (17)
C7	0.0521 (19)	0.0430 (19)	0.0470 (18)	0.0001 (14)	0.0107 (15)	−0.0006 (14)
C8	0.058 (2)	0.077 (3)	0.0484 (19)	0.0023 (17)	0.0103 (15)	0.0142 (16)
C9	0.056 (2)	0.088 (3)	0.062 (2)	0.0021 (18)	0.0107 (16)	0.0231 (19)
C10	0.0503 (18)	0.070 (2)	0.067 (2)	0.0038 (16)	0.0088 (16)	0.0188 (17)
C11	0.0472 (17)	0.049 (2)	0.0495 (18)	0.0049 (14)	0.0131 (14)	0.0086 (14)
C12	0.0428 (16)	0.049 (2)	0.0401 (17)	0.0037 (14)	0.0116 (13)	0.0050 (15)
C13	0.0522 (18)	0.048 (2)	0.0488 (18)	0.0073 (15)	0.0074 (14)	0.0112 (14)
C14	0.0546 (19)	0.047 (2)	0.058 (2)	0.0111 (15)	0.0157 (15)	0.0076 (15)
C15	0.104 (3)	0.070 (3)	0.068 (2)	0.001 (2)	0.039 (2)	0.0161 (18)
C16	0.067 (2)	0.084 (3)	0.076 (2)	0.0130 (19)	−0.011 (2)	0.0042 (19)
C17	0.087 (3)	0.068 (3)	0.086 (3)	−0.015 (2)	0.024 (2)	−0.0091 (19)
C18	0.087 (3)	0.082 (3)	0.089 (3)	0.040 (2)	0.027 (2)	0.008 (2)

### Geometric parameters (Å, °)

**Table:**

Cl1—C1	1.738 (3)	C9—C10	1.513 (4)
O1—C7	1.229 (3)	C9—H9A	0.9700
O2—N2	1.283 (3)	C9—H9B	0.9700
O3—N3	1.279 (3)	C10—C11	1.535 (4)
N1—C7	1.346 (3)	C10—H10A	0.9700
N1—C8	1.466 (3)	C10—H10B	0.9700
N1—C11	1.467 (3)	C11—C12	1.484 (4)
N2—C12	1.336 (3)	C11—H11	0.9800
N2—C13	1.500 (4)	C13—C15	1.521 (4)
N3—C12	1.328 (3)	C13—C16	1.523 (4)
N3—C14	1.497 (4)	C13—C14	1.550 (4)
C1—C2	1.366 (4)	C14—C18	1.517 (4)
C1—C6	1.367 (4)	C14—C17	1.529 (4)
C2—C3	1.374 (4)	C15—H15A	0.9600
C2—H2A	0.9300	C15—H15B	0.9600
C3—C4	1.392 (4)	C15—H15C	0.9600
C3—H3A	0.9300	C16—H16A	0.9600
C4—C5	1.382 (4)	C16—H16B	0.9600
C4—C7	1.502 (4)	C16—H16C	0.9600
C5—C6	1.376 (4)	C17—H17A	0.9600
C5—H5	0.9300	C17—H17B	0.9600
C6—H6	0.9300	C17—H17C	0.9600
C8—C9	1.503 (4)	C18—H18A	0.9600
C8—H8A	0.9700	C18—H18B	0.9600
C8—H8B	0.9700	C18—H18C	0.9600
			
C7—N1—C8	129.7 (2)	H10A—C10—H10B	109.0
C7—N1—C11	118.8 (2)	N1—C11—C12	112.2 (2)
C8—N1—C11	111.4 (2)	N1—C11—C10	103.5 (2)
O2—N2—C12	125.7 (2)	C12—C11—C10	113.3 (2)
O2—N2—C13	122.0 (2)	N1—C11—H11	109.2
C12—N2—C13	111.9 (2)	C12—C11—H11	109.2
O3—N3—C12	125.4 (2)	C10—C11—H11	109.2
O3—N3—C14	122.2 (2)	N3—C12—N2	109.4 (2)
C12—N3—C14	112.1 (2)	N3—C12—C11	126.1 (3)
C2—C1—C6	121.2 (3)	N2—C12—C11	124.5 (3)
C2—C1—Cl1	119.8 (3)	N2—C13—C15	110.0 (2)
C6—C1—Cl1	119.0 (3)	N2—C13—C16	105.2 (2)
C1—C2—C3	119.4 (3)	C15—C13—C16	111.2 (3)
C1—C2—H2A	120.3	N2—C13—C14	100.4 (2)
C3—C2—H2A	120.3	C15—C13—C14	114.7 (3)
C2—C3—C4	120.8 (3)	C16—C13—C14	114.3 (3)
C2—C3—H3A	119.6	N3—C14—C18	108.5 (3)
C4—C3—H3A	119.6	N3—C14—C17	105.7 (2)
C5—C4—C3	118.2 (3)	C18—C14—C17	110.0 (3)
C5—C4—C7	123.9 (3)	N3—C14—C13	100.9 (2)
C3—C4—C7	117.7 (3)	C18—C14—C13	116.2 (3)
C6—C5—C4	121.1 (3)	C17—C14—C13	114.3 (3)
C6—C5—H5	119.5	C13—C15—H15A	109.5
C4—C5—H5	119.5	C13—C15—H15B	109.5
C1—C6—C5	119.3 (3)	H15A—C15—H15B	109.5
C1—C6—H6	120.4	C13—C15—H15C	109.5
C5—C6—H6	120.4	H15A—C15—H15C	109.5
O1—C7—N1	120.3 (3)	H15B—C15—H15C	109.5
O1—C7—C4	119.8 (3)	C13—C16—H16A	109.5
N1—C7—C4	120.0 (2)	C13—C16—H16B	109.5
N1—C8—C9	103.3 (2)	H16A—C16—H16B	109.5
N1—C8—H8A	111.1	C13—C16—H16C	109.5
C9—C8—H8A	111.1	H16A—C16—H16C	109.5
N1—C8—H8B	111.1	H16B—C16—H16C	109.5
C9—C8—H8B	111.1	C14—C17—H17A	109.5
H8A—C8—H8B	109.1	C14—C17—H17B	109.5
C8—C9—C10	103.4 (2)	H17A—C17—H17B	109.5
C8—C9—H9A	111.1	C14—C17—H17C	109.5
C10—C9—H9A	111.1	H17A—C17—H17C	109.5
C8—C9—H9B	111.1	H17B—C17—H17C	109.5
C10—C9—H9B	111.1	C14—C18—H18A	109.5
H9A—C9—H9B	109.1	C14—C18—H18B	109.5
C9—C10—C11	103.9 (2)	H18A—C18—H18B	109.5
C9—C10—H10A	111.0	C14—C18—H18C	109.5
C11—C10—H10A	111.0	H18A—C18—H18C	109.5
C9—C10—H10B	111.0	H18B—C18—H18C	109.5
C11—C10—H10B	111.0		
			
C6—C1—C2—C3	0.4 (5)	C14—N3—C12—C11	173.7 (2)
Cl1—C1—C2—C3	−179.5 (2)	O2—N2—C12—N3	178.9 (2)
C1—C2—C3—C4	0.9 (4)	C13—N2—C12—N3	−9.2 (3)
C2—C3—C4—C5	−1.3 (4)	O2—N2—C12—C11	−1.8 (4)
C2—C3—C4—C7	−176.6 (3)	C13—N2—C12—C11	170.2 (2)
C3—C4—C5—C6	0.4 (4)	N1—C11—C12—N3	49.2 (4)
C7—C4—C5—C6	175.4 (3)	C10—C11—C12—N3	−67.6 (4)
C2—C1—C6—C5	−1.3 (5)	N1—C11—C12—N2	−130.0 (3)
Cl1—C1—C6—C5	178.6 (2)	C10—C11—C12—N2	113.2 (3)
C4—C5—C6—C1	0.9 (5)	O2—N2—C13—C15	−46.3 (3)
C8—N1—C7—O1	−175.4 (3)	C12—N2—C13—C15	141.3 (2)
C11—N1—C7—O1	0.4 (4)	O2—N2—C13—C16	73.5 (3)
C8—N1—C7—C4	4.3 (4)	C12—N2—C13—C16	−98.8 (3)
C11—N1—C7—C4	−179.9 (2)	O2—N2—C13—C14	−167.6 (2)
C5—C4—C7—O1	−135.9 (3)	C12—N2—C13—C14	20.1 (3)
C3—C4—C7—O1	39.1 (4)	O3—N3—C14—C18	−45.1 (4)
C5—C4—C7—N1	44.4 (4)	C12—N3—C14—C18	141.5 (3)
C3—C4—C7—N1	−140.7 (3)	O3—N3—C14—C17	72.9 (3)
C7—N1—C8—C9	156.1 (3)	C12—N3—C14—C17	−100.5 (3)
C11—N1—C8—C9	−20.0 (3)	O3—N3—C14—C13	−167.8 (3)
N1—C8—C9—C10	35.2 (3)	C12—N3—C14—C13	18.9 (3)
C8—C9—C10—C11	−37.6 (3)	N2—C13—C14—N3	−21.4 (3)
C7—N1—C11—C12	57.7 (3)	C15—C13—C14—N3	−139.2 (3)
C8—N1—C11—C12	−125.8 (3)	C16—C13—C14—N3	90.6 (3)
C7—N1—C11—C10	−179.8 (2)	N2—C13—C14—C18	−138.5 (3)
C8—N1—C11—C10	−3.3 (3)	C15—C13—C14—C18	103.7 (3)
C9—C10—C11—N1	25.2 (3)	C16—C13—C14—C18	−26.5 (4)
C9—C10—C11—C12	147.0 (3)	N2—C13—C14—C17	91.5 (3)
O3—N3—C12—N2	179.9 (3)	C15—C13—C14—C17	−26.3 (4)
C14—N3—C12—N2	−7.0 (3)	C16—C13—C14—C17	−156.4 (3)
O3—N3—C12—C11	0.6 (5)		

### Hydrogen-bond geometry (Å, °)

**Table:**

*D*—H···*A*	*D*—H	H···*A*	*D*···*A*	*D*—H···*A*
C2—H2A···O2^i^	0.93	2.54	3.458 (4)	170
C3—H3A···O1^i^	0.93	2.45	3.314 (4)	154
C8—H8A···O3^ii^	0.97	2.44	3.101 (4)	125

Symmetry codes: (i) −*x*+1, −*y*, −*z*; (ii) −*x*+3/2, *y*−1/2, −*z*+1/2.
